# Echoes of the Month

**Published:** 1923-02

**Authors:** 


### ECHOES OF THE MONTH.

### MINISTER OF HEALTH ON SMALLPOX.

Writing in the " Sunday Express " on the Health of 1922, Sir Arthur Griffith-Boscawen says that the recent visitation of smallpox should serve as a warning. " Great Britain, though an island and itself virtually free from smallpox, is in these days of new-fashioned transport in closer touch than ever with overseas countries which have not yet succeeded in stamping out smallpox from their midst. For instance, a person who contracts infection in Cairo or New York can travel to and land in England, and not show signs of disease until he has reached his home. So long as this disease exists anywhere we shall be liable at any time to have a case in this country. No administrative medical net has a mesh fine enough to guarantee us against the importation of the infection, and so long as this is so it is the duty of the nation and its health authorities to be prepared. This leads me to a vexed question, I know, but it is the vastly preponderating opinion of the world's medical men that in vaccination lies the highest degree of safety from smallpox, and it is a national health duty to walk and work in the light of that fact."

### GOOD BREATHING AND GOOD THINKING.

" Deep breathing and high thinking " is the version of Wordsworth's famous formula suggested by Dr. Alfred Mumford, the Medical Officer of Manchester Grammar School, in an exceedingly interesting address to the Ling Association in connection with the recent Educational Conference at University College. Dr. Mumford was giving the results of some of the records which he has been keeping for the past twelve years. In that time he has had opportunity for the methodical study of the physical condition of some 4,000 boys, and these school tests have gone to show that the " brainy " boy is also the boy with the efficient physical apparatus. Boys who came to the school with scholarships have " clearer respiratory passages " and better teeth than the average, and he asserts a real connection between good breathing and good thinking.

### HOSPITAL FIRES.

It is startling to learn, says the *Journal of the American Medical Association*, that, during 1919 and 1920, 870 fires occurred in institutions classified as hospitals, asylums and sanatoriums—an average of more than one fire a day in that type of institution. If any structures should be properly protected, those in which human beings lie incapable of self-protection should be given first consideration. Among the causes of fire in these institutions were, first, sparks on the roof; second, defective chimneys and flues; third, lightning; fourth, heating installation; and fifth, electricity. These etiologic considerations point the way to prevention, but there are further special considerations which apply to hospitals. The height of hospital buildings is a matter for concern; ambulatory patients should be placed on the higher floors, the lower floors being reserved for those completely bedridden. In every hospital there should be located fire alarm boxes for prompt notification in case of fire. Volatile liquids which are easily inflammable should be handled with caution. Chemical extinguishers should be easily available, and a fire drill for the employees should be a regular experience. " When it is realised," says the National Board of Fire Underwriters, "that most fires are preventable, and that there are none too many hospitals, it seems a pity that large sums must go annually toward replacing what might have been conserved for further usefulness."

### DOCTORS' REWARDS AND RISKS IN BABYLON.

Lecturing to the Brighton and Hove Archæological Club on " The Medicines of the Ancient Empires of Babylonia and Assyria," Dr. L. A. Parry, F.R.C.S., said that the physicians in those vanished empires were sufficiently advanced to vary their fees according to the capacity of the patient to pay, one of their laws being to the effect that " The doctor who treats a man for a grievous wound with a bronze knife and heals him, or cuts the film (probably meaning a cataract) of the man with a bronze knife and heals it, shall receive ten shekels of silver. But if the patient be a poorer man he shall receive five shekels, and only two if he is a slave." If, on the other hand, a patient died or lost his sight under this treatment, the surgeon would have both his hands cut off! " I shudder to think," said Dr. Parry, " how few doctors would be alive if such a law were in force at the present time." Four thousand years ago the Assyrians were concerned in passing laws for the protection of nurse-children, the difference between the ideas of justice at that time and the present being illustrated, however, by a law that if a man caused a woman's death by miscarriage his daughter should be put to death. Doctors to-day recognised for use in medicine many drugs the names of which were found inscribed in the arrow-headed cruciform writing on the clay tablets of ancient Assyria. Having regard to the fact that these ancient doctors had very little medical knowledge to guide them, they reached a high state of medical culture which one could not fail to admire, while there was ample evidence that the native races had more than an elementary knowledge of therapeutics and nomenclature of diseases.

### GOOD AND BAD AUTO-SUGGESTION.

That stammering and nervous diseases can be cured by auto-suggestion was the claim put forward by Dr. William Brown, Wilde Reader in Mental Philosophy, University of Oxford, in an address to the National League for Health, Maternity, and Child Welfare. Dr. Brown had an amusing story to tell of how M. Coué, on a visit which he paid to Oxford, showed himself keenly alert to kill bad auto-suggestions " as if they were flies." He was pressed to make a good breakfast, as he was about to make a journey. He objected that it was the train and not he which was going to make the journey. Dr. Brown considered it a bad auto-suggestion when a person who felt a twinge of indigestion believed he was going to be really ill. A good auto-suggestion would be that the attack would soon pass off. He described the method by which he effected a particular cure. The person was told to lie down and think of sleep. Then his forehead was stroked, and the suggestion was made that the trouble would not occur again. Fear and mental conflicts in childhood were considered by the lecturer to be the causes of many of the things which auto-suggestion would cure, and the general lesson, in his view, was that as far as possible a child should be allowed to develop along its own lines and in accordance with its own interests. When it was necessary to check a child the process should be made rational by giving a reason, and not merely saying it must not be done.

### LEASEHOLD PROPERTY AND PUBLIC HEALTH.

In a paper read before the Auctioneers' and Estate Agents' Institute, Mr. T. P. Bennett, F.R.I.B.A., expressed regret for many reasons, that obligatory town-planning scheme, are in the hands of local authorities. No system of development by public authorities will ever equal a system of good development carried out by private owners, the strong personal incentive which actuates the private owner being absent. He knows that the exponents of the individualistic system instance cases in which development carried out by private owners has been derogatory to all concerned; that is due to the lack of proper direction on the part of professional advisers. It is in the interest of the community that the maximum amount of building should take place. It is an argument in favour of the leasehold system that it is a system under which rebuilding is most likely to come about. By it buildings revert to landlords who in many cases are indisposed to collect rents or manage the property; therefore they cast about for fresh owners, and endeavour to create fresh leases. Furthermore, the property tends to revert to one landlord in blocks, instead of in separate entities, and it is possible to consider fresh schemes in accordance with altered conditions. As regards the charge that leasehold encourages the erection of buildings which last a bare 99 years, it will be found in most cases that, with the advance of modern conditions, far more buildings have been destroyed because these conditions have rendered them obsolete than because the lease has expired or the building decayed. The interference of any public body in this question, such as that involved in condemning property as insanitary or dangerous, or otherwise forcing owners to rebuild, operates with the maximum amount of disturbance and friction, while the leasehold system probably obtains quite a desirable end with the minimum amount of disturbance and friction.

### PURE BUT COSTLY.

At the conference of the University Labour Federation at Liverpool, Dr. S. G. Moore, Medical Officer of Huddersfield, contended that human life was purchasable, and within limits the community could determine its own death-rate. If we were to take all the precautions necessary for securing clean milk the inevitable result was that milk was going to cost a lot more. The remedy was Pasteurization and a higher price paid for milk of the highest quality instead of a rate which now obtained. Milk supply should be taken over by the municipality. A resolution was passed declaring, *inter alia*, that the milk supply ought ultimately to be municipalised.

### THE RED CROSS IN THE AIR.

A fleet of aerial ambulances, each to accommodate four stretcher cases and two attendants, has been completed for the use of the Royal Air Force. Machines of a similar type, but larger and more commodious, are under construction. Every possible convenience for the patients has been introduced into the craft, and travel in them is as comfortable as in any ground ambulance. The machines are equipped with surgical devices of a kind that might be of avail when a soldier or airman was being rushed at more than two miles a minute to hospital. There is a filter to ensure a pure supply of water, tubes of compressed oxygen, and the apparatus for its administration and a hand-basin with taps leading from water storage tanks. Attention is being given to the production of a flying surgical operating theatre. The first of the aerial ambulances are intended for use in countries like Irak, where a crash may occur in the desert, far remote from any hospital or any town where attention can be given to an injured soldier.

### THE STERILISATIO\ OF MENTAL DEFECTIVES.

At a meeting of the Eugenics Education Association, Professor E. W. McBride, Professor of Biology in the Imperial College of Science, gave an address on "Mental Defect and its Inheritability." He said that the problem with which they had to deal was not peculiar to the human race. If they cultivated any large quantity of plants or raised any large number of domestic animals, sooner or later these deviations from the normal, these defects, would appear. So denunciations of our social system as the cause of mental defect were unscientific. The lower-grade mental defectives were maintained in institutions and did not propagate themselves, but the higher-grade defectives mingled with the general population. They gravitated to the slums and bred recklessly. If slums were cleared away and these people were put in ideally constructed dwellings they would reproduce the slums again in 30 or 40 years. There was only one remedy, which he did not expect to see applied in this country, though they were already beginning in America. That was the ruthless sterilisation of mental defectives, so that they would not be able to hand on these defects to posterity.

### STUDYING NON-PULMONARY TUBERCULOSIS.

The report recently issued by Dr. Lissant Cox, the Central Tuberculosis Officer of the Lancashire County Council, mentions a particularly sensible arrangement in connection with the equipment of the Tuberculosis Officers for the county. In order that they might keep themselves conversant with the latest methods of diagnosis and treatment of non-pulmonary tuberculosis, especially in children, arrangements were made at the end of 1921 whereby each tuberculosis officer in turn would be appointed honorary clinical assistant to Sir Henry Gauvain at the Lord Mayor Treloar Cripples' Hospital and College, Alton, Hants. At the date of Dr. Lissant Cox's report, five of the senior officers had already completed their course, and a sixth was then there. The experience so gained had been most valuable, and the officers were placed in a much better position to deal with the number of non-pulmonary cases referred to them by practitioners for diagnosis and treatment. The methods of treatment of this form of tuberculosis have undergone considerable changes during the past few years, and are greatly in advance of those in vogue some seven or eight years ago. Conspicuous progress has been made in recent years in the treatment of crippled children, and, although the number of these cases which are afforded institutional treatment by the County Council is very small, the general claims made as to the effectiveness of the new methods receive confirmation in the fact that of the 94 children treated 64 have been certified fit for school.

### INCIDENCE OF SUICIDE.

An interesting monograph on suicide from the international point of view has just been produced by Dr. Miner, of the School of Hygiene and Public Health, Johns Hopkins University. The main fact revealed is that the incidence of the suicide rate varies widely and in a most surprising manner in different countries. For instance, Germany, France, Denmark and Sweden possess the highest rate, while in Great Britain, Norway, the Netherlands, and the South and East of Europe comparatively low rates prevail. Again, as contrasted with this country, the United States has a much higher rate. Suicides are far commoner in towns than in country districts, and much more frequent among men than among women. As showing the effects of marital responsibility, the rate is stated to be far lower among married than among single or widowed people. Although the Germans have a particularly high rate and the Slav nations have one that is correspondingly low, the reason for this state of things, according to the best judges, is to be found not so much in racial as in religious differences, for it is well established that suicide is more frequent in Protestant communities than in those which belong to the Roman Catholic or the Orthodox Greek Church.

### BUILDING OUR OWN HOUSES.

In the January *Contemporary Review* Mr. Ernest Betham describes a scheme by which the small man may build his own house. The initial requirement would be sectionalised and archtecturally prepared plans of several sorts of one-story bungalow, giving the various stages of construction in plain and practicable sequence. The plans should provide for a substantial but simple house, fulfilling the right standard of design and complying with the requirements of the Public Works Loan Board and the bye-laws, and they should be drawn and described in a way understandable by the layman, technicalities being translated into the vernacular. They should be sectionalised in a manner which would allow of the completion of the first half of a house as a self-contained unit. This would include the living room, a bedroom, water supply and drainage, so that a man, beginning building in spring, could live in the first half before autumn, and finish the erection under more favourable conditions than having to journey to and fro in the evenings and at week-ends. Together with the consecutive sets of working drawings there would be supplied printed directions for each stage, put in plain language, so that a man would be able to proceed with the work as surely as would an intelligent child with a building of toy bricks when each measurement, stage and brick was indicated. Either from the surveyor of the Local Authority, or other qualified person, guidance would be obtainable when required, and it would be understood from the outset that matters of water supply and sanitation should be under the supervision of experts. No house could be occupied until the customary certificate of habitation from the local authority had been issued. Nothing more than the loans from the Public Works Loan Board normally obtainable under the Housing Acts should be called on ; neither subsidy nor private capital would be wanted. The margin between the Public Works Loan Board's advance of two-thirds and the value of the houses would be covered by the labour value.

### "CANCER HOUSES."

Dr. William J. Simpson, C.M.G., Professor of Hygiene at King's College, writes:—"The question of 'cancer houses' should be investigated again. I am aware of a striking instance in which cancer appeared in members of three families in succession occupying a cottage. They were not related. The cottage was pulled down by the landlord. Strangely, a case of cancer has recently appeared in an adjoining detached cottage."

